# A Multi-Center Study on Sensitization to Thimerosal in North-Eastern Italy, 1997–2023: Prevalence, Risk Factors, the Role of Occupation and the Impact of Vaccinations

**DOI:** 10.3390/vaccines13060622

**Published:** 2025-06-09

**Authors:** Luca Cegolon, Emilia Patriarca, Francesca Larese Filon

**Affiliations:** 1 Department of Medical, Surgical & Health Sciences, University of Trieste, 34128 Trieste, Italy; emilia.patriarca@asugi.sanita.fvg.it (E.P.); larese@units.it (F.L.F.); 2 Public Health Unit, University Health Agency Giuliano-Isontina (ASUGI), 34128 Trieste, Italy; 3 Occupational Medicine Unit, University Health Agency Giuliano-Isontina (ASUGI), 34128 Trieste, Italy

**Keywords:** Thimerosal, Methiolate, sensitization, allergic contact dermatitis, patch test, vaccination, prevalence, standard series, allergology

## Abstract

**Background**: Thimerosal has been widely used as a preservative to prevent microbial growth in medications and vaccines. However, in 1999 its removal from vaccine formulations was called for due to concerns about its potential side effects on humans, with subsequent reduced sensitizations at patch tests. The present multi-center study investigated the epidemiological, occupational and temporal pattern of sensitization to Thimerosal in North-Eastern Italy during 1997–2023 and associated factors. **Methods**: Due to variability in patch testing and positive reactions by the centers, this study was broken down by three periods: 1997–2004 (including all centers but Trieste); 1997–2015 (considering only Padua and Pordenone); and 2010–2023 (considering only Trieste and Pordenone). Multiple logistic regression was used to investigate prevalence of sensitization to Thimerosal and associated factors. Results were expressed as adjusted odds ratio (aOR) with 95% confidence intervals (95%CI). **Results**: Prevalence of positive patch test reactions to Thimerosal decreased from (8.13%) in 1997 to 0.95% in 2023 across all centers combined. Prevalence of positivity to Thimerosal was 9.49% during 1997–2004 (in all centers yet excluding Trieste), 8.41% during 1997–2015 (considering only Padua and Pordenone) and 4.01% during 2010–2023 (considering only Trieste and Pordenone). A significantly decreasing trend of Thimerosal sensitization was observed during 1997–2015 (aOR = 0.94; 95%CI: 0.92; 0.95). Regardless of the study period, sensitization to Thimerosal was consistently and significantly higher among health care workers (HCWs) and in patients born during 1981–1990. **Conclusions**: The significantly decreasing prevalence of sensitization to Thimerosal over time likely reflected removal policies from vaccines and medications after 1999. Likewise, the higher prevalence of patch test reactions in patients born during 1981–1990 may mirror the widespread presence of this hapten in vaccines and medications in the 1980ies. Moreover, the increased prevalence of patch test reactions positive to Thimerosal in HCWs probably reflected higher influenza vaccination uptake in this group compared to other occupational categories. Positive patch test reactions to Thimerosal after 2000 were likely clinically irrelevant though.

## 1. Introduction

### 1.1. Thimerosal in Human Vaccines and Medications

Thimerosal (Methiolate) is a mercury derivative composed of ethyl mercury chloride (EtHgCl) and thiosalicylic acid (TSA), developed in 1927 as a preservative with anti-microbial properties [1,2,3].

Thimerosal has been employed since the 1930ies as a preservative in a range of medications, including topical antiseptic solutions and ointments to treat wounds, nasal sprays, eye solutions, vaginal spermicides and diaper rash treatment [2]. Thimerosal has also been widely used in childhood vaccines, especially to prevent microbial growth in multi-dose vials formulations [4,5,6].

Global skepticism on health (neurological) side effects of Thimerosal as an additive to medications started to spark from the early 1980ies, leading to its removal from all vaccines in Denmark in 1992 [7]. Likewise, in Sweden, all childhood vaccines were already Thimerosal-free by 1993 [8]. In particular, Thimerosal-containing childhood vaccinations were indicted to increase the risk of neuro-developmental disorders such as autism, attention-deficit/hyperactivity disorder and speech delay [9].

On 7 July 1999, the US Public Health Service (USPHS, including the Food and Drug Administration, the National Institutes of Health, the Center for Disease Control and Prevention and Health Resources and Services Administration) and the American Academy of Pediatrics (AAP) called for removal of Methiolate from all vaccines as soon as possible as a precautionary principle [5,10], leading to its progressive elimination from novel and existing vaccine formulations in Europe and North America [3,5,11], with the exceptions of multi-dose influenza vaccines [12] and vaccines available in developing countries [3].

Although no evidence of harm associated with vaccinations was available, the USPHS was concerned that cumulative exposure to mercury by childhood immunizations may have exceeded the limit set by the Environmental Protection Agency’s (EPA) for methylmercury [10]. The EPA had in fact decreased the recommended maximum limit of exposure to methyl-mercury allowed for humans from 0.5 mcg/Kg to 0.1 mcg/kg per day [13]. Until 1999, all vaccines against tetanus, diphtheria-tetanus-acellular pertussis (DTPs), Haemophilus influenza type b (Hib), hepatitis B (HBV) and polysaccharide meningococcal meningitis ACWY contained Thimerosal, often at 0.01% concentrations [3]. A vaccine including 0.01% Thimerosal contains approximately 25 mcg of mercury per dose [6]. More than 20 vaccines authorized in the USA in 2001 included Thimerosal at concentrations ranging from 0.003% to 0.01% [14].

Safety concerns on Thimerosal were therefore based on evidence regarding the detrimental effects of methyl-mercury, whereas recipients of vaccines and medications containing Thimerosal are exposed to ethyl-mercury [8]. A study conducted in Denmark on 956 children diagnosed with autism during 1971–2000 in fact found no evidence of increased risk of autism at the time when Thimerosal was included in vaccines, and the incidence increased and continued to rise even after its removal from vaccines, including increases among children born after its discontinuation [7]. Likewise, an ecological study conducted in Sweden, Denmark and California found no evidence of increased risk of autism associated with Thimerosal-containing vaccines between mid-1980s and late-1990s [8]. However, some authors argued that Thimerosal is not an optimal preservative for vaccines at currently used dosage formulations, and higher concentrations would reduce vaccine efficacy as well as increasing the risk of side effects in recipients [3].

Given the open debate, the World Health Organization (WHO) issued the following official position: “*It is important to note that concerns about the toxicity of Thiomersal are theoretical and that there is no compelling scientific evidence of a safety problem related to its use in vaccines, although public perception of risk has been reported in some countries*” [15].

Likewise, following a 18-month review on risks and benefits of Thimerosal, the European Agency for the Evaluation of Medicinal Products (EMEA) issued the following statement: “*Although there is no evidence of harm caused by the level of exposure from vaccines, it would be prudent to promote the general use of vaccines without thimerosal … within the shortest possible time-frame*” [16]. Moreover, the EMEA recommended to display a label on all Thimerosal-containing vaccines, warning patients on the risk of sensitization, which was first reported in 1999 [17].

### 1.2. Thimerosal as a Hapten

Immune-mediated reactions to mercury-containing products are well documented in humans and in experimental animals, the most frequent being allergic contact dermatitis (ACD) [18,19].

Animal models suggested that Thimerosal may induce dermatitis either by pseudo-allergic reactions (a particular form of irritant dermatitis involving mast cells) or delayed-type hypersensitivity response. However, it is unclear whether the latter two mechanisms co-exist in the same individual allergic to Thimerosal [20].

Thimerosal was appointed allergen of the year in 2002 by the American Contact Dermatitis Society, due to a high prevalence of sensitization combined with low clinical relevance (<7.2%) [19,21]. Sensitization to Thimerosal increased from 6.2% in 1984 to 10% in 2002 in North America, with a plateau in the mid-1990s, not hinting at a reduced use in vaccines over time [22].

Although susceptible individuals immunized when Thimerosal-containing vaccines were still circulating may have developed life-long sensitization [18], this hapten was removed from several standard patch test screening series. Since it is now one of the less relevant allergens causing ACD as result of its removal from most vaccines, sensitization to Thimerosal has been progressively decreasing over time and probably destined to extinguish and/or to be considered clinically irrelevant [19,23].

Most clinically relevant allergic reactions to Thimerosal occur following cosmetics use [24,25,26] or after contact with ophthalmic preparations, resulting mostly in facial dermatitis [19,27]. Consequently, Thimerosal sensitivity is more frequent in females due to cosmetic use and in occupational categories as cooks or health care workers (HCWs) due to exposure to the hapten through vaccinations [19,28].

In view of the above, the present multi-center study investigated the epidemiological, occupational and temporal pattern of sensitization to Thimerosal in North-Eastern Italy during 1997–2023 and associated factors.

## 2. Methods

Prevalence of sensitization to Thimerosal was investigated in 31,948 consecutive patients patch tested for suspected ACD during 1997–2023 (27 years) in four centers of Triveneto (North-Eastern Italy)—Trieste, Padua, Pordenone and Bolzano/Trento/Rovigo—to identify potential trends and associated factors. This study was approved by the local ethical committee of Friuli Venezia Giulia (CEUR, protocol 092/2018), and written informed consent was obtained from all participating patients.

### 2.1. Evaluation of Patients and Patch Testing

The clinical pattern of patients was assessed using the MOAHLFA Index (considering sex of patient, occupational dermatitis, atopic dermatitis, hand dermatitis (HD), leg dermatitis (LD), face dermatitis (FD), age > 40 years), which was consistently applied in all allergology centers [29].

Occupation was classified using ISCO-88 codes and then summarized in groups with similar exposure.

Occupational dermatitis was assessed by a dermatologist or an occupational medicine consultant, considering the clinical history, sites involved, occupational exposures and stop and go test.

All patients were patch tested with Finn Chambers (Epitest, Tuusula, Finland) on Scanpor tape (Norgesplaster, Vennesla, Norway) and haptens produced by Chemotechnique Diagnostics (Vellinge, Sweden) and by FIRMA (Florence, Italy). During the overall period, European baseline series and the extended Triveneto series (Supplementary Table S1) were used to patch test patients for suspected ACD. Thimerosal was tested in petrolanum (pet.) at 1% in all centers except in Trieste, where a concentration of 0.01% was used from 1997 to 2009, subsequently updated to 0.1% from 2010 onward.

All patches were applied on the upper part of patient’s back and removed after 48 h. The area was examined upon removal of the patch (D2) and after 72/96 h (D3/D4), according to guidelines of the International Contact Dermatitis Research Group [30]:

- Reactions degree +, ++ and +++ were considered positive.
- Doubtful reactions (?+) were considered negative.

### 2.2. Statistical Analysis

Since Trento–Bolzano–Rovigo contributed only until 2004, Padua stopped testing Thimerosal after 2015, and in Trieste the formulation used for patch test was tenfold diluted (0.01% pet.) during 1997–2009, the prevalence of sensitization to was assessed in these time periods:

- Years 1997–2004 in all centers but Trieste;
- Across 1997–2015, limited to Padua and Pordenone;
- Across 2010–2023, limited to Trieste and Pordenone.

The year of birth of patients patch tested was split into categories in order to take into account the effect of various vaccination policies enforced in Italy over time. In particular, birth years were broken down as follows [31]:

- **1904–1938**: when only vaccination against small pox was mandatory in Italy since 1888.
- **1939–1965**: since diphtheria vaccination was mandatorily introduced in 1939.
- **1966–1980:** since polio vaccination was enforced in 1966 and tetanus in 1968.
- **1981–1990**: since small pox immunization was abolished in 1981.
- **1991–1998:** since HBV vaccination was made mandatory for all newborns.
- **1999 onward**: when a call for removal of Thimerosal from all new vaccine formulations was launched in USA.

Continuous variables were presented as mean and standard deviation as well as median and interquartile range (IQR). Medians were compared by Mann–Whitney test, whereas the Chi-squared test was employed to compare categorical variables.

Factors associated with prevalence of sensitization to Thimerosal were investigated by three multiple logistic models, structured as follows:

- **Model 1:** across 1997–2004, including all centers but Trieste;
- **Model 2:** across 1997–2015, limited to Padua and Pordenone;
- **Model 3:** across 2010–2023, limited to Trieste and Pordenone.

Backward stepwise procedure (*p* < 0.05) was employed to build multivariable logistic regression models. Results were expressed as adjusted odds ratio (aOR) with 95% confidence interval (95%CI). The Benjamini–Hochberg selection (setting the false discovery rate at 5%) was finally applied to exclude false positive results.

Statistical analysis was performed with STATA version 14.0 (Stata, College Station, TX, USA).

## 3. Results

**Figure 1:**
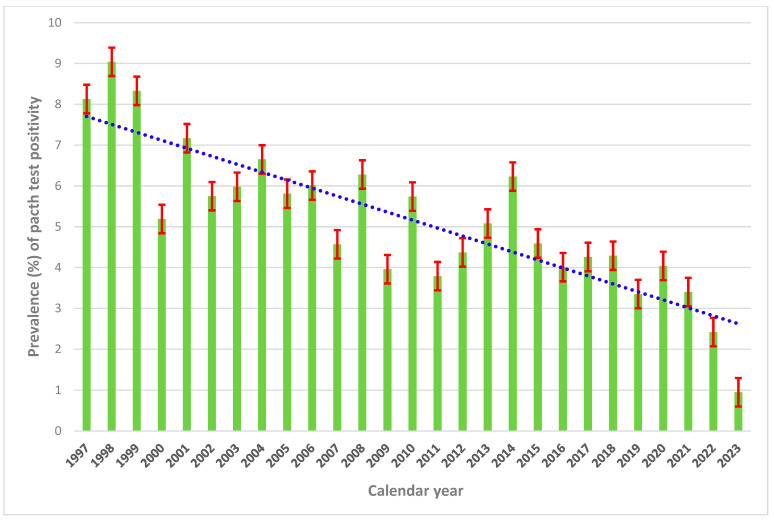
Prevalence (%) of patch test positivity to Thimerosal in all centers combined, 1997–2023.

A variability of testing and sensitization was observed in all centers combined, with a decreasing time trend from (8.13%) in 1997 to 0.95% in 2023 (Supplementary Table S2 and Figure 1).

**Figure 2:**
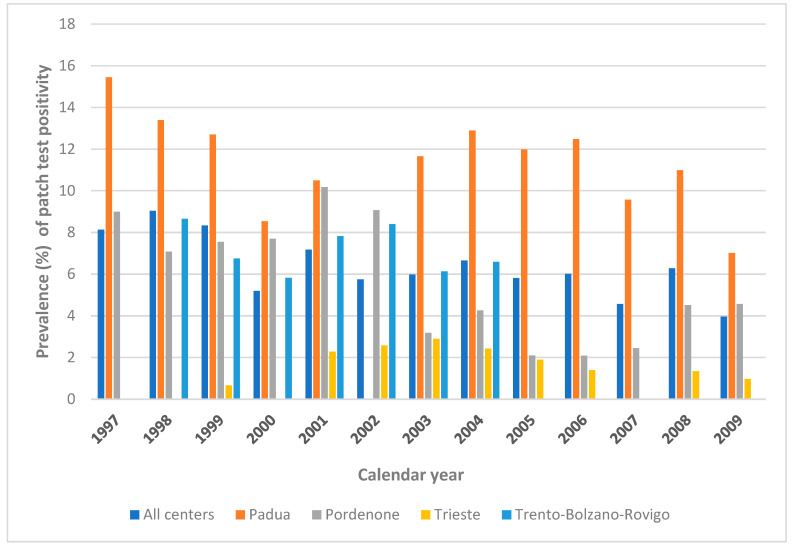
Prevalence (%) of patch test positive reactions to Thimerosal by center, 1997–2009.

**Figure 3:**
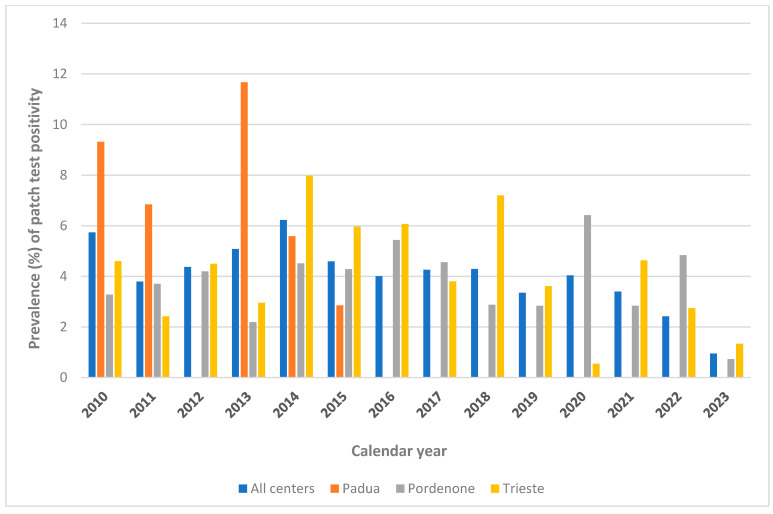
Prevalence (%) of patch test positive reactions to Thimerosal by center, 2010–2023.

The distribution of positive patch test reactions by research center can be viewed in Figure 2 (years 1997–2009) and Figure 3 (years 2010–2023).

Table 1 displays the distribution of age by calendar year of birth. As it can be seen, 79.82% patients born during 1939–1945 were patch tested at the age of 43+ years, whereas 83.98% of those born in 1966–1980 were patch tested at the age ≤ 42 years, 79.87% of patients born during 1981–1990 were patch tested before the age of 30 and 97.41% patients born in 1990–1998 were patch tested at < 30 years of age (Table 1).

Table 2 displays the descriptive distribution of the study population by sensitization to Thimerosal and explanatory factors, broken down by study period (1997–2004 in all centers but Trieste vs. 1997–2015, considering only Padua and Pordenone vs. 2010–2023, limited to Pordenone and Trieste). Positive patch test results dropped in patients born after 1998—with zero positive reactions in those born after 2000.

Prevalence of positive patch test reactions to Thimerosal decreased from 9.49% during 1997–2004 (in all centers but Trieste) to 8.41% during 1997–2015 (considering only Padua and Pordenone) and to 4.01% during 2010–2023 (considering only Trieste and Pordenone) (Table 2).

Sensitization to Thimerosal in Trieste increased from 1.31% during 1997–2009 to 4.19%, after updating the respective patch test formulation from 0.01% pet. to 0.1% pet.

### 3.1. Calendar Years 1997–2004 (Considering All Centers but Trieste)

As mentioned above, the overall prevalence of positive reactions to Thimerosal during 1997–2004 was 9.49% (in all centers but Trieste), higher in Padua (11.84%) and lower in Trento–Bolzano–Rovigo (7.40%) (Table 2).

The median age of patients patch tested during 1997–2004 was 38 years, being 33 years among those testing positive to Thimerosal, and there was no difference in sensitization by sex (*p* = 0.780). The prevalence of female sex among patients sensitized to Thimerosal in 1997–2004 was 67.79% (=705/1040).

Among patients patch tested during this period, sensitization to Thimerosal progressively increased from 2.80% in patients born during 1904–1938 to 8.95% in those born during 1939–1965, 10.43% in those born 1966–1980 and 23.02% in the birth cohort 1981–1990 (Table 2).

The body area most frequently affected by dermatitis during 1997–2004 was the face (N = 3938), followed by hands (N = 1735) and legs (N = 637) (Table 2).

During 1997–2004, the prevalence of atopic dermatitis was 4.44% among patients undergoing a patch test and 6.14% among those positive to Thimerosal. During these years, the prevalence of occupational dermatitis was 9.68% (=1061/10,962) among patients undergoing a patch test and 12.21% (=127/1040) among those positive to Thimerosal (Table 2).

Table 3 (upper panel) displays multiple logistic regression analysis on factors associated with sensitization to Thimerosal during 1997–2004. Prevalence of Thimerosal sensitization during 1997–2004 decreased over time (aOR = 0.95; 95%CI: 0.91; 0.98), was significantly higher in Padua (aOR = 1.69; 95%CI: 1.40; 2.04) and increased in patients born during 1939–1965 (aOR = 2.49; 95%CI: 1.72; 3.62), 1966–1980 (aOR = 2.74; 95%CI: 1.85; 4.06) and especially in the birth cohort 1981–1990 (aOR = 8.13; 95%CI: 5.28; 12.51). Furthermore, compared to clerks, sensitization to Thimerosal was significantly higher in HCWs (aOR = 1.50; 95%CI: 1.22; 1.83) and lower in retirees (aOR = 0.62; 95%CI: 0.42; 0.92) and housewives (aOR = 0.77; 95%CI: 0.60; 0.99), despite the latter two estimates were dropped at BH selection (Table 3).

### 3.2. Calendar Years 1997–2015 (Considering Only Padua and Pordenone)

The overall prevalence of Thimerosal positivity during 1997–2015 was 8.41%, higher in Padua (10.79%) than Pordenone (4.97%) (Table 2).

The median age of patients testing positive to Thimerosal during 1997–2015 was 40 years (33 years among patients testing positive) without a difference in sensitization by sex (*p* = 0.157). Prevalence of female sex among patients sensitized to Thimerosal in 1997–2015 was 68.54% (=841/1227) (Table 2).

In patients patch tested during 1997–2015, sensitization to Thimerosal increased from 2.67% in those born during 1904–1938 to 7.37% in those born during 1939–1965, 9.18% in those born 1966–1980, and 17.14% in birth cohort 1980–1990, decreasing to 4.62% in those born during 1991–1998 and 0% among patients born after 1998 (Table 2).

The body areas most frequently affected by dermatitis in patients patch tested during 1997–2015 were again the hands (N = 5097), followed by face (N = 2974) and legs (N = 1046) (Table 2).

During 1997–2015, prevalence of atopic dermatitis was 8.94% among patients undergoing a patch test and 8.65% (=105/1214) among those positive to Thimerosal. During the same period, prevalence of occupational dermatitis was 6.54% among patients patch tested and 8.39% (=103/1227) among those positive to Thimerosal (Table 2).

Table 3 (middle panel) displays multiple logistic regression analysis on factors associated with sensitization to Thimerosal during 1997–2015. A significantly decreasing time trend of patch test-positive reactions to Thimerosal could be observed during 1997–2015 (aOR = 0.94; 95%CI: 0.92; 0.95). Moreover, prevalence of Thimerosal sensitization was significantly higher in Padua (aOR = 2.01; 95%CI: 1.73; 2.33) than Pordenone and, compared to patients born during 1904–1938, increased among those born in 1939–1965 (aOR = 2.52; 95%CI: 1.78; 3.58), in 1966–1980 (aOR = 2.98; 95%CI: 2.07; 4.31) and especially 1980–1990 (aOR = 8.17; 95%CI: 5.56; 12.01), decreasing in birth cohort 1991–1998 (aOR = 2.89; 95%CI: 1.55; 5.40) (Table 3, middle panel).

Sensitization to Thimerosal was again significantly more prevalent in HCWs (aOR = 1.45; 95%CI: 1.18; 1.77) and lower in retirees (aOR = 0.62; 95%CI: 0.43; 0.88) and cleaners (aOR = 0.13; 95%CI: 0.02; 0.92), although the latter estimate was dropped at BH selection (Table 3).

### 3.3. Calendar Years 2010–2023 (Considering Only Trieste and Pordenone)

The overall prevalence of patch test-positive reactions to Thimerosal during 2010–2023 reduced to 4.01%, being higher in Trieste (4.19%) than Pordenone (3.78%) (Table 2).

The median age of patients testing positive to Thimerosal during 2010–2023 was 45 years (37 among those testing positive), and there was a significantly lower prevalence of sensitization in males (3.37%) compared to females (4.09%; *p* = 0.043) (Table 2). Prevalence of female sex among patients sensitized to Thimerosal in 2010–2023 was 73.27% (=89/333).

Sensitization to Thimerosal increased from 1.25% in patients born during 1904–1938 to 2.12% in those born 1939–1965, 5.02% in those born during 1966–1980, and 8.68% in birth cohort 1981–1990, decreasing to 3.07% among those born in 1991–1998 and to 1.33% in patients born during 1999–2015 (Table 2).

In this period, the prevalence of atopic dermatitis was 18.12% among patients undergoing a patch test and 24.55% (=81/330) among those positive to Thimerosal.

Prevalence of occupational dermatitis was 9.31% among patients undergoing a patch test and 13.51% (=45/333) among those positive to Thimerosal (Table 2).

The body areas most frequently affected by dermatitis during 2010–2023 were consistently the hands (N = 2539), followed by face (N = 1716) and legs (N = 785) (Table 2).

Table 3 (lower panel) displays multiple logistic regression analysis on factors associated with sensitization to Thimerosal during 2010–2023. Prevalence of positive reactions was significantly higher among HCWs (aOR = 1.84; 95%CI: 1.28; 2.64) and birth years 1981–1990 (aOR= 3.59; 95%CI: 1.19; 10.82), although the latter estimate failed to pass BH selection. Other significant factors excluded at BH selection were face dermatitis (aOR = 1.37; 95%CI: 1.06; 1.78), chemistry workers (aOR = 3.22; 95%CI: 1.19; 8.67), retirees (aOR = 0.49; 95%CI: 0.26; 0.95), unemployed (aOR = 0.40; 95%CI: 0.17; 0.94) and restaurant workers (aOR = 0.51; 95%CI: 0.27; 0.97) (Table 3).

## 4. Discussion

### 4.1. Prevalence of Sensitization

With variability by center and calendar year, prevalence of Thimerosal sensitization reduced from 1997 (8.13%) through 2023 (0.95%), significantly decreasing during 1997–2015. Average sensitization was 9.49% during 1997–2004 (in all centers but Trieste) versus 8.41% across 1997–2015 (considering only Padua and Pordenone) and 4.01% during 2010–2023 (considering only Pordenone and Trieste).

Prevalence of sensitization to Thimerosal in Trieste increased from 1.31% during 2010–2023 to 4.19% after the respective patch test formulation was updated from 0.01% to 0.1% pet.

Thimerosal has reportedly been one of the most frequent sensitizers over the years. Already back in 1972–74, among 3000 patients patch tested in North America, Thimerosal was among the 19 most common reacting allergens, along with nickel sulfate, caine mixture, potassium dichromate, balsam of Peru, ethylenediamine hydrochloride, paraphenylenediamine and thiram [32]. Thimerosal was therefore recommended to be included in standard series of North America.

As mentioned above, removal of Thimerosal from all vaccines was called for in 1999 by some major medical associations from North America and Europe [5,10,33]. Residual use of mercury, including in vaccines, was subsequently targeted on 24 June 2005 by the Council of the European Union [34], integrating the community strategy of the European Commission concerning mercury issued on 28 January 2005 [35]. On 14 March 2006, the European Parliament resolution prompted the European Commission for a restriction on Thimerosal use, with the ultimate goal to fully ban it when safe alternatives existed, supporting also research on Thimerosal-free multi-dose vaccine formulations [36].

After some initial resistance to implement policies against Thimerosal-containing vaccines [37], European member states anticipated the above European directive though. For instance, in France all childhood vaccines were Thimerosal-free already by 2002, apart from two HBV vaccines [38]. The Department of Health of the UK announced that Thimerosal would be removed from all childhood vaccinations in August 2004 [39]. In Italy, a governmental decree issued on 13 November 2001 ordered the replacement of all Thimerosal-containing vaccines by 30 June 2003, unless no alternatives existed [40].

As a likely result of the above policies, sensitization to Thimerosal in 2591 patients patch tested in Europe during 2015–2018 dropped to 2.51% [41]. Nonetheless, higher prevalence of sensitization was recently reported in North America. For instance, among 3767 patients patch tested during 2010–2023 by the Mayo Clinic Contact Dermatitis Group (USA), prevalence of Thimerosal sensitization was 9.3% [42]. Likewise, among 38,482 NACDG patients patch tested during 2001–2016, the most common allergens in both adults and children with atopic dermatitis were in fact nickel sulfate, methyl-isothiazolinone, formaldehyde, fragrance mix I, sodium gold thiosulfate and Thimerosal 0.1% pet. [43]. In particular, among 36,834 adult patients (32.6% males), the prevalence of Thimerosal sensitization was 9.1% in those with versus 10.2% in those without history of atopic dermatitis. Among 1648 children <18 years of age, the latter percentages increased to 15.6% in those with versus 19.0% in those without a history of atopic dermatitis [41]. Thimerosal is reportedly still used as a preservative in some vaccines in the USA, and this may explain the higher prevalence of positive reactions compared to Europe. In particular, flu vaccines are currently available both as Thimerosal-containing (for multi-dose vials) and Thimerosal-free formulations [44]. Although, the latter discrepancy in sensitization prevalence may also reflect higher use of Thimerosal in vaccines and medications in USA compared to Europe in the past.

Mercury and related compounds were already banned from cosmetics items by the Cosmetics Directive 76/768/EEC. Removal of Thimerosal from medical products in the European Union was further reiterated by the Cosmetic Regulation No. 1223/2009. Mercury-containing compounds are allowed only in eye products and with a maximum concentration not exceeding 0.007% mercury when used alone or in combination with other mercurial compounds. Additionally, the labelling “*Contains Thiomersal*” or “*Contains Phenylmercuric compounds*” needs to be displayed on mercury-containing eye formulations [45].

In contrast to nickel, the elimination of Thimerosal from cosmetic products and ophthalmic medications has led to a significant reduction in positive patch test responses among patients with periorbital dermatitis [46]. For instance, a single-center study from the University Hospital of Siena (Tuscany, central Italy) conducted during 1997–2021 on 7955 patients patch tested for suspected ACD reported a marked decline in Thimerosal patch test positivity over time, likely reflecting reduced use of the hapten in ophthalmic formulations [46]. By contrast, sensitization to nickel exhibited an opposite trend in the latter study [46].

Although Thimerosal sensitization was associated with face dermatitis during 2010–2023 in the present study, the respective positive reactions were again likely irrelevant, in light of the above removal policies of this preservative from topical products after 30 June 2003 in Italy [2].

### 4.2. The Impact of Vaccination

Regardless of the study period (1997–2004 vs. 1997–2015 vs. 2010–2023), sensitization to Thimerosal was consistently higher in patients born during 1981–1990, a likely reflection of widespread use of Methiolate as a medical preservative in the 1980s [2,3]. By contrast, the drop in sensitizations in patients born during 1991–1998, when HBV vaccination was enforced in Italy, may reflect growing concerns about Methiolate across Europe following its ban from vaccines in Denmark in 1992 [7]. Positive patch test reactions in the present study, therefore, likely reflected sensitization triggered by Thimerosal-containing childhood vaccinations. Likewise, the further drop of sensitizations in patients born after 1998—with zero positive reactions in those born after 2000—could be explained by removal policies of Methiolate from all new vaccine formulations in Europe. The use of mercury-containing preservatives has in fact declined in recent years, due to development of new single-dose vaccine formulations not requiring preservatives. Other preservatives currently authorized by the Food and Drug Administration (FDA) are Phenol (included in vaccines to prevent pneumococcal disease, typhoid fever, monkey pox and small pox), 2-Phenoxyethanol (included in polio vaccination) and Benzethonium chloride (in vaccines against Bacillus Anthracis) [6].

Despite information on vaccination status at the individual level and vaccination coverage by region and calendar years was not available in the present study, the above childhood vaccinations (diphtheria, tetanus, pertussis, polio, HBV) were mandatory in Italy; hence, the majority of Italian citizens born during the 1980s were likely immunized, as confirmed by the high (~95%) and relatively stable DTP uptake reported in Italy for the years 2000–2024 [47]. Exposure to medications containing Methiolate may have also contributed to induce sensitization, but this information was also not available in the present study.

Regardless of the study period, the role of vaccination was also endorsed by the significantly and consistently higher prevalence of sensitization to Thimerosal among HCWs, who in addition to childhood immunizations, are also more likely to be immunized against influenza for professional reasons. At least until 2008, several influenza vaccines authorized in the European Union in fact still reportedly contained Thimerosal [12]. The decreased significance of prevalence of patch test reactions in patients born during 1981–1990 and patch tested during 2010–2023 may reflect progressively waning sensitization to the hapten with increasing time since childhood immunizations. By contrast, the consistently higher prevalence of sensitization to Thimerosal in HCWs patch tested during 2010–2023 may be induced by immunizations with Thimerosal-containing influenza vaccines received after 2000. Likewise, the consistently lower prevalence of sensitization in retirees in all study periods may reflect lower immunogenicity associated with increasing age as well as increasing time since childhood vaccinations.

The higher prevalence of positive reactions in Padua during the years 1997–2004 and the years 1997–2015 may be explained by higher uptake for childhood immunizations in this area, since stronger vaccine hesitancy is consistently reported in Trentino-Alto Adige (where Trento and Bolzano are located) [48]. However, this is a hypothesis that needs further research to be confirmed. During 1997–2004, prevalence of positive reactions in Trento–Bolzano–Rovigo (7.40%) was comparable to Pordenone (7.53%), hence a similar declining trend may have been expected in both centers over time, should have Trento–Bolzano–Rovigo contributed after 2004.

Although a Thimerosal allergy is typically most common among females due to cosmetic use [19,28], no association was found by the sex of patients in all three regression models.

### 4.3. Strength and Weaknesses

The present is the largest multi-center study investigating Thimerosal sensitization in Italy over a long time period (27 years), assessing also the impact of occupation. Since Thimerosal has been removed from most standard series, this study is one of the few providing information on sensitization to this hapten in patch tests performed after 2000.

Study limitations include the cross-sectional design, the variability of testing rate by research center and calendar year and the lack of a relevance definition for Thimerosal sensitization. However, avoiding assessment of clinical relevance, as done in other epidemiological studies [49,50,51,52], averts interpretation biases.

The vast majority of patients patch tested during 1997–2023 in the present study were probably not exposed to the hapten, and positive reactions were likely unrelated to their actual dermatitis, but rather with sensitization to Thimerosal triggered before 1999, hence before its removal from vaccines and medications in Europe and North America.

Since the start of data collection, the reading of patch tests was performed for the majority of patients at 96 h. The reading at 72 h was performed in Trieste in about a fourth of patients due to organizational and logistic constraints. However, patients were still recommended to come back to be seen in ambulatory in the event of late reactions onset. We believe therefore this bias had a marginal impact on study findings and is unlikely to explain the lower prevalence of positive reactions observed in Trieste before 2010. After 2009, the prevalence of positive patch tests reactions in Trieste (4.19%) were in fact even higher than Pordenone (3.78%), endorsing the hypothesis that the main factor explaining lower sensitization in Trieste before 2010 was a less reactive patch test formulation.

Although information on vaccination status at the individual level and vaccination coverage by birth year and region was not available, the above vaccinations were mandatory in Italy, and around 95% Italians were reportedly immunized against DTP during 2000–2023. Therefore, Thimerosal positivity likely reflected sensitization to the hapten through childhood vaccinations or (in case of HCWs) also to influenza, although exposure to medications may have also contributed.

## 5. Conclusions

The prevalence of Thimerosal sensitization was 5.89% across the entire study period (1997–2023), decreasing from 1997 (8.13%) through 2023 (0.95%) in all centers combined, reflecting progressively lower use of this preservative in vaccine formulations and cosmetic products over time.

Positive patch test reactions peaked in patients born during 1981–1990, when vaccines and medications containing Methiolate were probably more widespread. Sensitizations in these patients may have thus been triggered by Thimerosal-containing childhood vaccinations. Likewise, the higher prevalence of sensitization in HCWs may be explained by higher uptake of Thimerosal-containing influenza vaccinations in this group compared to other occupations. However, positive reactions to Thimerosal in patients patch tested after 2000 are likely clinically irrelevant.

## Figures and Tables

**Table 1:** Distribution of calendar year of birth by age of patients at patch tests. Median, interquartile range (IQR); number and row percentage (%).

Calendar Year of Birth	Patients’ Age at Patch Test (Years)				
	Median (IQR)	N (%)			
		<30	30–42	43–57	58+
**1904–1938**	72 (67; 77)	0	0	0	3383 (100)
**1939–1965**	52 (44; 59)	0	2600 (20.18)	6240 (48.43)	4045 (31.39)
**1966–1980**	32 (27; 39)	4110 (41.39)	4278 (43.09)	1541 (15.52)	0
**1981–1990**	24 (19; 29)	2651 (79.87)	668 (20.13)	0	0
**1991–1998**	21 (18; 25)	1205 (97.41)	32 (2.59)	0	0
**1999–2015**	18 (16; 21)	446 (100)	0	0	0

**Table 2:** Study population by patch test results against Thimerosal.

**TERMS**			**Period 1997–2004** **(Padua, Pordenone, Trento–Bolzano–Rovigo)**			**Period 1997–2015** **(Padua and Pordenone)**			**Period 2010–2023** **(Trieste and Pordenone)**		
			**Total Tested (N)**	**Thimerosal +** **N (Row %)**	***p*** **-** **Value**	**Total Tested (N)**	**Thimerosal +** **N (Row %)**	***p*** **-** **Value**	**Total Tested (N)**	**Thimerosal +** **N (Row %)**	***p*** **-Value**
**Total Patients Examined for Suspected ACD**			**10,962**	**1040 (9.49)**		**14,596**	**1227 (8.41)**		**8305**	**333 (4.01)**	
**Center**	**Padua**		5085	11.84	<0.001	8619	10.79	<0.001	-	-	0.347
	**Pordenone**		2243	7.53		5976	4.97		3700	3.78	
	**Trieste**		-	-		-	-		4605	4.19	
	**Trento/Bolzano/Rovigo**		3633	7.40		-	-		-	-	
**Sex**	**Females**		7388	9.54	0.780	9737	8.64	0.156	5665	4.31	0.043
	**Males**		3573	9.38		4858	7.95		2640	3.37	
**Age** (years) (M: 4)	**M (SE)**		41.10 (0.16)	34.2 (0.42)		42.6 (0.14)	34.8 (0.39)		45.7 (0.19)	39.6 (0.73)	
	**Median (IQR)**		38 (28; 53)	33 (23; 43)	<0.001 *	40 (29; 55)	33 (24; 44)	<0.001 *	45 (32; 59)	38 (30; 48)	<0.001 *
	**≤40**		5813	12.16	<0.001	7114	11.41	<0.001	3228	5.45	<0.001
	**41+**		5148	6.47		7481	5.55		5076	3.09	
**Birth year**	**1904–1938**		1537	2.80	<0.001	1613	2.67	<0.001	320	1.25	<0.001
	**1939–1965**		4858	8.95		6106	7.37		3019	2.12	
	**1966–1980**		3884	10.43		4804	9.18		2429	5.02	
	**1981–1990**		682	23.02		593	17.14		1279	8.68	
	**1991–1998**		-	-		433	4.62		879	3.07	
	**1999–2015**		-	-		45	0		377	1.33	
**Atopic dermatitis** (M: 3140)	**No**		8856	9.85	0.008	13,134	8.44	0.713	6710	3.71	0.002
	**Yes**		412	13.83		1289	8.15		1485	5.45	
**Occupational** **dermatitis** (M: 31)	**No**		9900	9.22	0.004	13,641	8.24	0.006	7504	3.84	0.007
	**Yes**		1061	11.97		954	10.80		770	5.84	
**Body area affected** **by dermatitis**	**Hand** (M: 4432)	**No**	5273	9.63	0.551	8798	8.24	0.573	5530	3.87	0.102
		**Yes**	3938	10.01		5097	8.51		2539	4.65	
	**Leg** (M: 4430)	**No**	8574	9.80	0.957	12,849	8.34	0.977	7286	4.24	0.079
		**Yes**	637	9.73		1046	8.32		785	2.93	
	**Face** (M: 4430)	**No**	7476	9.59	0.176	10.921	8.35	0.936	6355	3.84	0.017
		**Yes**	1735	10.66		2974	8.31		1716	5.13	
**Occupation** **(M: 65)**	**Clerks**		2428	12.11	<0.001	2852	10.38	<0.001	1595	4.70	<0.001
	**Health care workers**		1641	14.20		1397	14.03		680	8.53	
	**Teachers**		-	-		126	8.73		312	5.13	
	**Cashiers**		-	-		12	8.33		16	6.25	
	**Sellers**		-	-		145	3.45		222	6.76	
	**Restaurant workers**		478	12.13		520	9.81		470	2.34	
	**Hairdressers**		98	8.16		116	7.76		131	3.82	
	**Farmers**		139	12.23		128	10.16		46	2.17	
	**Construction workers**		682	10.85		556	9.35		193	3.63	
	**Painters**		12	8.33		40	10.00		48	10.42	
	**Mechanics**		528	8.52		685	6.13		481	4.78	
	**Workers of wood industry**		218	8.26		291	6.87		100	4.00	
	**Artisans general**		335	7.16		270	8.15		34	8.82	
	**Leather artisans**		94	7.45		81	7.41		5	0	
	**Chemistry workers**		146	13.01		155	11.61		36	13.89	
	**Drivers**		161	7.45		148	9.46		53	3.77	
	**Cleaners**		95	6.32		126	0.79		107	2.80	
	**Housewives**		1831	6.01		1700	6.06		711	3.52	
	**Students**		-	-		421	5.23		605	3.14	
	**Retirees**		1326	3.39		1809	2.76		1232	1.14	
	**Unemployed**		198	10.10		304	8.22		323	1.86	
	**Other**		551	8.89		2713	9.80		881	3.86	

* = Mann–Whitney test *p*-value. Number (N), column and row percentage (%), chi square *p*-value, mean with standard error (SE), median with interquartile range (IQR). ACD = Allergic contact dermatitis; M = missing values. Thimerosal positivity calculated out of total tested.

**Table 3:** Multiple logistic regression analysis for the sensitization to Thimerosal. Adjusted odds ratio (aOR) with 95% confidence interval (95%CI). Benjamini–Hochberg (BH) *p*-value set at 5% false discovery rate. NS = non-significant. HCW = health care workers. Significant estimates at BH selection are yellow marked for aOR > 1 or green marked for aOR < 1; in both cases **the darker the colour the higher the effect size**.

Study Period	Terms		aOR (95%CI)	*p*- Value	BH *p* Value
**Years** **1997–2004** **(Padua, Pordenone, Trento–Bolzano–Rovigo)** (10,962 obs.)					**BH *p* ≤ 0.0130**
	**Center**	**Padua**	1.69 (1.40; 2.04)	<0.001	0.0022
		**Pordedone**	reference		
		**Trento–Bolzano–Rovigo**	1.00 (0.82; 1.23)	0.976	-
	**Birth year**	**1904–1938**	reference		
		**1939–1965**	2.49 (1.72; 3.62)	<0.001	0.0043
		**1966–1980**	2.74 (1.85; 4.06)	<0.001	0.0065
		**1981–1990**	8.13 (5.28; 12.51)	<0.001	0.0087
		**1991–1998**	omitted		
		**1999–2015**	omitted		
	**Occupation**	**Clerks**	reference		
		**HCWs**	1.50 (1.22; 1.83)	<0.001	0.00130
		**Housewives**	0.77 (0.60; 0.99)	0.045	0.0174 (NS)
		**Retirees**	0.62 (0.42; 0.92)	0.018	0.0152 (NS)
	**Calendar year (1997–2004)—linear term**		0.95 (0.91; 0.98)	0.003	0.0130
**Years** **1997–2015** **(Limited to Padua and Pordenone)** (14,550 obs.)					**BH *p* ≤ 0.0148**
	**Center**	**Padua**	2.01 (1.73; 2.33)	<0.001	0.0037
		**Pordedone**	reference		-
	**Birth year**	**1904–1938**	reference		
		**1939–1965**	2.52 (1.77; 3.58)	<0.001	0.0056
		**1966–1980**	2.98 (2.07; 4.31)	<0.001	0.0074
		**1981–1990**	8.17 (5.56; 12.01)	<0.001	0.0093
		**1991–1998**	2.89 (1.55; 5.40)	0.001	0.0130
		**1999–2015**	-	-	-
	**Occupation**	**Clerks**	reference		
		**HCWs**	1.45 (1.18; 1.77)	<0.001	0.0011
		**Cleaners**	0.13 (0.02; 0.92)	0.041	0.0167 (NS)
		**Retirees**	0.62 (0.43; 0.88)	0.007	0.0148
	**Calendar year (1997–2015)—linear term**		0.94 (0.92; 0.95)	<0.001	0.0019
**Years** **2010–2023** **(limited to Pordenone and Trieste)** (7936 obs.)					**BH *p* ≤ 0.0019**
	**Center**	**Trieste**	1.12 (0.88; 1.42)	0.346	-
		**Pordedone**	reference		
	**Face** **dermatitis**	**No**	reference		
		**Yes**	1.37 (1.06; 1.78)	0.036	0.0179 (NS)
	**Atopic** **dermatitis**	**No**	reference		
		**Yes**	1.29 (0.98; 1.68)	0.065	-
	**Birth year**	**1904–1938**	reference		
		**1939–1965**	1.03 (0.35; 2.99)	0.962	-
		**1966–1980**	1.95 (0.65; 5.84)	0.235	-
		**1981–1990**	3.59 (1.19; 10.82)	0.023	0.0071 (NS)
		**1991–1998**	1.33 (0.41; 4.29)	0.632	-
		**1999–2015**	0.67 (0.16; 2.89)	0.593	-
	**Occupation**	**Clerks**	reference		
		**HCWs**	1.84 (1.28; 2.64)	0.001	0.0018
		**Restaurant workers**	0.51 (0.27; 0.97)	0.041	0.0143 (NS)
		**Chemistry workers**	3.22 (1.19; 8.67)	0.021	0.0054 (NS)
		**Retirees**	0.49 (0.26; 0.95)	0.035	0.0089 (NS)
		**Unemployed**	0.40 (0.17; 0.94)	0.036	0.0089 (NS)
	**Calendar year (2010–2023)—linear term**		0.97 (0.94; 1.00)	0.036	0.0107 (NS)

## Data Availability

The data generated and analyzed during the current study are not publicly accessible, but they may be available from the corresponding author upon reasonable request.

## Supplementary Materials

The following supporting information can be downloaded at: https://www.mdpi.com/article/10.3390/vaccines13060622/s1, Supplementary Table S1: Triveneto patch test series (22 haptens) tested in the overall study period, all in petrolanum (pet.) when not otherwise specified. Supplementary Table S2: Frequency distribution of patients patch tested for contact dermatitis and rates of positivity against para-tertiary-butylphenol-formaldehyde resin (PTBP-FR), by calendar year (1997–2021) and research centre. Number (N) and row percentage (%); Supplementary Table S3: Frequency distribution of patients patch tested for contact dermatitis and rates of positivity against para-tertiary-butylphenol-formaldehyde resin (PTBP-FR), by calendar year (1997–2021) and research centre. Number (N) and row percentage (%).
