# Supplementary material for: A Multi-Center Study on Sensitization to Thimerosal in North-Eastern Italy, 1997–2023: Prevalence, Risk Factors, the Role of Occupation and the Impact of Vaccinations
Source: Vaccines (Basel). 2025 Jun 9;13(6):622. doi: 10.3390/vaccines13060622 (PMC12197333; doi:10.3390/vaccines13060622)
Supplement: Supplementary file 1 [file vaccines-13-00622-s001.zip › vaccines-3633327-supplementary.pdf]

Supplementary Table S1. Triveneto patch test series (22 haptens) tested in the overall study period, all in petrolanum (pet.) when not otherwise specified.

**1**	4-ter-Butylphenol- formaldehyde resin 1%
**2**	Carba mix 3%
**3**	Cobalt chloride hexahydrate 1%
**4**	Colophonium 20%
**5**	Disperse blu 35 1%
**6**	Disperse yellow 3 1%
**7**	Epoxy resin 1%
**8**	Formaldehyde 1% aq
**9**	Fragrance mix-I 8%
**10**	Methyl-chloro-isothiazolinon/methyl-isothiazolinon (Kathon) 0.02 aq.
**11**	Lanolin alcohol 30%,
**12**	Mercaptobenzothiazole 2%
**13**	Mercaptobenzothiazole mix 2%
**14**	Neomycin sulfate 20%
**15**	Nickel sulphate 5%
**16**	N-Isopropy-N-phenyl-4-phenylendiamine 0.1%
**17**	Parabens mix 16%
**18**	Peru balsam 25%
**19**	Potassium bichromate 0.5%
**20**	p-Phenylenediamine 1%
**21**	Quaternium-15 1%
**22**	Thiuram mix 1%

Supplementary Table S2. Frequency distribution of patients patch tested for contact dermatitis and rates of positivity against Thimerosal by calendar year (1997-2021) and research centre. Number (N) and row percentage (%)

CALENDAR YEAR	ENTIRE COHORT		RESEARCH CENTRE							
			Padua		Pordenone		Trieste		Trento/Bolzano/Rovigo	
	N. tests	Thimerosal +	N. tests	Thimerosal +	N. tests	Thimerosal +	N. tests	Thimerosal +	N. tests	Thimerosal +
**1997**	1,242	101 (8.13)	395	61 (15.44)	445	40 (8.99)	398	0	4	0
**1998**	2,566	232 (9.04)	1,083	145 (13.39)	325	23 (7.08)	418	0	740	64 (8.65)
**1999**	2,868	239 (8.33)	1,189	151 (12.70)	278	21 (7.55)	453	3 (0.66)	948	64 (6.75)
**2000**	2,445	127 (5.19)	1,042	89 (8.54)	208	16 (7.69)	817	0	378	22 (5.82)
**2001**	2,412	173 (7.17)	677	71 (10.49)	226	23 (10.18)	703	16 (2.28)	806	63 (7.82)
**2002**	1,460	84 (5.75)	-	-	353	32 (9.07)	701	18 (2.57)	405	34 (8.40)
**2003**	1,472	88 (5.98)	412	48 (11.65)	314	10 (3.18)	485	14 (2.89)	261	16 (6.13)
**2004**	842	56 (6.65)	287	37 (12.89)	94	4 (4.26)	370	9 (2.43)	91	6 (6.59)
**2005**	1,135	66 (5.81)	434	52 (11.98)	383	8 (2.09)	318	6 (1.89)	-	-
**2006**	1,032	62 (6.01)	409	51 (12.47)	336	7 (2.08)	287	4 (1.39)	-	-
**2007**	1,115	51 (4.57)	439	42 (9.57)	368	9 (2.45)	308	0	-	-
**2008**	1,257	79 (6.28)	528	58 (10.98)	355	16 (4.51)	374	5 (1.34)	-	-
**2009**	1,112	44 (3.96)	328	23 (7.01)	373	17 (4.56)	411	4 (0.97)	-	-
**2010**	1,046	60 (5.74)	354	33 (9.32)	366	12 (3.28)	326	15 (4.60)	-	-
**2011**	1,107	42 (3.79)	234	16 (6.84)	377	14 (3.71)	496	12 (2.42)	-	-
**2012**	664	29 (4.37)	-	-	286	12 (4.20)	378	17 (4.50)	-	-
**2013**	886	45 (5.08)	240	28 (11.67)	274	6 (2.19)	372	11 (2.96)	-	-
**2014**	1,011	63 (6.23)	322	18 (5.59)	288	13 (4.51)	401	32 (7.98)	-	-
**2015**	958	44 (4.59)	246	7 (2.85)	327	14 (4.28)	385	23 (5.97)	-	-
**2016**	749	30 (4.01)	-	-	239	13 (5.44)	280	17 (6.07)	-	-
**2017**	399	17 (4.26)	-	-	241	11 (4.56)	158	6 (3.80)	-	-
**2018**	723	31 (4.29)		-	451	13 (2.88)	250	18 (7.20)	-	-
**2019**	627	21 (3.35)	-	-	211	6 (2.84)	416	15 (3.61)	-	-
**2020**	446	18 (4.04)	-	-	265	17 (6.42)	181	1 (0.55)	-	-
**2021**	500	17 (3.40)	-	-	176	5 (2.84)	266	12 (4.63)	-	-
**2022**	495	12 (2.42)	-	-	62	3 (4.84)	307	9 (2.74)	-	-
**2023**	633	6 (0.95)	-	-	137	1 (0.73)	412	5 (1.34)	-	-
**Total**	**31,202**	**1,837 (5.89)**	**9,163**	**930 (10.15)**	**7,758**	**366 (4.72)**	**10.648**	**272 (2.55)**	**3,333**	**269 (7.40)**

Supplementary Table S3. Frequency distribution of patients patch tested for contact dermatitis and rates of positivity against para-tertiary-butylphenol-formaldehyde resin (PTBP-FR), by calendar year (1997-2021) and research centre. Number (N) and row percentage (%)

CALENDAR YEAR	ENTIRE COHORT		RESEARCH CENTRE							
			Padua		Pordenone		Trieste		Trento/Bolzano/Rovigo	
	N. tests	Thimerosal +	N. tests	Thimerosal +	N. tests	Thimerosal +	N. tests	Thimerosal +	N. tests	Thimerosal +
**1997-2009**	20,958	1,402 (6.69)	7,224	828 (11.46)	4,058	226 (5.57)	6,043	79 (1.31)	3,633	269 (7.40)
**2010-2023**	10,957	434 (4.25)	2,629	102 (5.26)	3,700	140 (3.78)	4,628	193 (4.19)	NA	NA
